# Haplotype-resolved genome of a citronella provides insights into the evolution of citronelloid biogenesis pathway

**DOI:** 10.1093/hr/uhaf287

**Published:** 2025-10-16

**Authors:** Hai He, Zemian Lin, Zanchen Zhou, Pinhao Chen, Lifeng Xia, Hao Li, Yu Zhang

**Affiliations:** School of Agriculture andd Biotechnology, Sun Yat-Sen University, Shenzhen, China; School of Agriculture andd Biotechnology, Sun Yat-Sen University, Shenzhen, China; School of Agriculture andd Biotechnology, Sun Yat-Sen University, Shenzhen, China; School of Agriculture andd Biotechnology, Sun Yat-Sen University, Shenzhen, China; School of Agriculture andd Biotechnology, Sun Yat-Sen University, Shenzhen, China; School of Agriculture andd Biotechnology, Sun Yat-Sen University, Shenzhen, China; School of Agriculture andd Biotechnology, Sun Yat-Sen University, Shenzhen, China

Dear Editor,

Many plants employ citronellol for pollination and interorganism communication, while some leaves utilize citronellal for biotic defense [1, 2]. The defensive metabolites produced and stored constitutively in plant tissues are typically referred as phytoanticipins. This study focuses on natural citronelloid-related compounds (citronelloid refers to related oxygenated, acyclic monoterpenes like citronellal, citronellol, and geraniol) in the Poaceae family, specifically the *Cymbopogon* genus. *C. winterianus* is characterized by an abundant of volatile linear monoterpene alcohols and aldehydes. These compounds are biologically and economically significant, and have been utilized in cancer treatments to reduce cancer progression [3]. However, the detailed description of the monoterpenes biosynthesis mechanisms in *Cymbopogon* are unclear, and lack of genomic information hindered evolutionary studies and genome-based researches in citronelloid biosynthesis pathways.

*C. winterianus* is characterized by abundant citronellal and citronellol, while *C. distans* lack these compounds. The genomic information of the *Cymbopogon* genus offers valuable genetic resources for breeding and essential oil production. Hence, *C. winterianus* (Fig. 1a) and *C. distans* (Fig. 1b) were used to assemble from the *Cymbopogon* genus that have distinct ploidy levels. In total, we presented reference genomes for *C. winterianus* (diploid cultivar, 1.49 Gb) and *C. distans* (tetraploid wild species, 2.58 Gb) using PacBio sequencing in HiFi and Hi-C data, and 107 480/185756 predicted coding genes were identified, respectively. An overview of the genes is presented in Fig. 1c, such as the density distribution of genes, GC content and repeat sequences. We employed BUSCO analysis to assess the integrity of the assembled genomes. The results identified that the completeness of genome assembly was 99.10% for *C. winterianus* and 99.10% for *C. distans*. Consequently, we successfully completed two sets of haplotype-resolved chromosome-level genome sequences of *Cymbopogon*. These achievements lay a solid foundation for subsequent analyses.

**Figure 1:**
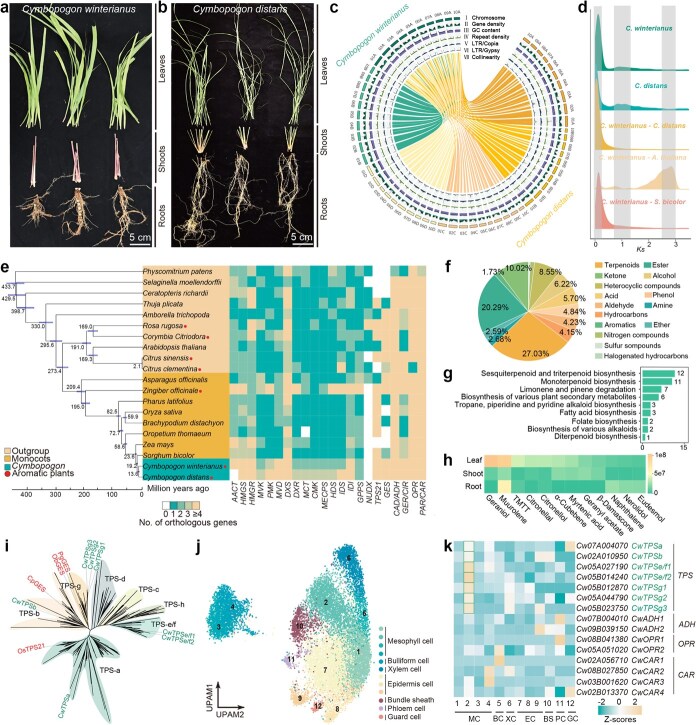
The multi-omics analysis of citronelloid biogenesis pathway in *Cymbopogon*. The plant division and individual fractions of leaves, shoots, and roots in *C. winterianus* (**a**) and *C. distans* (**b**) for multi-omics sampling. **c**. Overview of genomic features in *C. winterianus* and *C. distans*. From outside to inside: Track I illustrates genomic landscape of *C. winterianus* pseudomolecules (left) and *C. distans* pseudomolecules (right). Tracks II-VI, represented by five concentric rings, illustrate the density distribution of genes, GC content, repeat sequences, LTR/Copia, and LTR/Gypsy elements. Track VII, displays syntenic blocks between *C. winterianus* and *C. distans*. **d**. Analysis of *Ks* for paralogs and orthologs in *C. winterianus*, *C. distans*, and two additional species. The Y-axis represents genes frequency at the respective *Ks* values. **e**. Phylogenetic tree constructed from orthogroups across 21 plant genomes (the outgroup *Chlamydomonas reinhardtii* is not shown). Node support was calculated using 1000 bootstraps, with all nodes exhibiting support values exceeding 0.99. The numbers next to nodes represented the divergence times (million years ago), and the bars indicated the 95% confidence intervals for the divergence times between different clades. Adjacent heat maps illustrate the distribution of terpenoid biosynthesis pathway gene families among species. **f**. Pie chart illustrating the distribution of various metabolite categories. **g.** KEGG pathway enrichment of differential metabolites. **h**. The concentrations of the ten most abundant terpenes in leaf, shoot, and root tissues of *C. winterianus* were quantified utilizing GC–MS with three biological replicates. **i.** Phylogenetic analysis of selected *TPS* genes from *C. winterianus*, *C. distans*, and other plant species. The clades were classified according to the *AtTPS* genes family categorization. The candidate *CwTPS* genes in *C. winterianus* that exhibit strong correlations with citronelloid biosynthesis are highlighted in green, whereas the reported genes from other species that participate in the citronelloid biosynthesis pathways are colored red. **j.** UMAP projection illustrating 12 cell clusters derived from gene expression matrices of high-quality cells. **k.** Heatmaps show relative expression levels of candidate genes participating in the citronelloid biosynthesis pathway.

To assess evolutionary relationships between *Cymbopogon* and other species, we conducted an analysis on the *Ks* values of orthologous gene pairs. The analysis revealed peaks at *Ks* = 0.26 for *C. winterianus*–*Sorghum bicolor* and at *Ks* = 2.83 for *C. winterianus*–*Arabidopsis thaliana*, respectively. Through intragenomic comparison (dot-plot analyses) of *C. winterianus*, we characterized the structure of ancient duplications based on analysis of 3907 genes in the haplotype A subgenome of *C. winterianus* (Fig. S1). Comparative analysis of chromosomes revealed strong collinearity between *S. bicolor*, and the subgenomes of *C. winterianus* and *C. distans*, indicating that their structures were highly similar and well conserved (Fig. S2). These findings indicated that *C. winterianus*, *C. distans,* and *S. bicolor* likely shared a common origin from the same WGD events. Additionally, we observed peaks at *Ks* = 0.11 for *C. winterianus*–*C. distans* (Fig. 1d). This deviation might stem from specific variations in evolutionary rates. Therefore, we conducted divergence time estimation and phylogenetic analysis divergence time estimation with 19 other sequenced plant species based on their representative evolutionary positions, including eight monocot plants and five aromatic plants (Fig. 1e). Phylogenomic analysis revealed that two *Cymbopogon* species were clustered together, and diverged from their close outgroup (*S. bicolor*) approximately 19.2 million years ago (Fig. 1e).

Furthermore, we investigated the evolution of terpenoid biosynthesis pathway gene families in green plants. Among the 22 orthologous gene clusters examined, most appear to have originated or been lost only a few times across the monocot family and other species (Fig. 1e; Table S1). The number of copies of several genes that are involved in early terpenoid biosynthesis, such as *HMGS*, *HMGR*, and *IDI*, was expanded in the Poaceae. Notably, Nudix hydrolase (*NUDX*) orthologous genes were most frequently present among several early-evolving species outside the Poaceae family, suggesting that *NUDX* genes were lost during genome evolution in the Poaceae family. In some plants such as rose petals, geraniol can be produced through GPP-specific RhNUDX rather than the terpene synthase enzyme [4]. As anticipated, the copy numbers of most terpene synthases (TPSs) were increased in *Cymbopogon* and aromatic plants (*Zingiber officinale* and *Corymbia citriodora*) compared to all other species.

Therefore, we analyzed volatiles from leaves, shoots, and roots and identified 1158 compounds in *C. winterianus* using HS-SPME/GC-MS (Table S2). The primary volatile organic compounds (VOCs) were terpenoids, esters, and heterocyclic compounds (Fig. 1f). Terpenoids constituted the largest proportion (27.03%) when compared against KEGG database, with monoterpenoids, sesquiterpenoids, and triterpenoids being the most abundant among the detected terpenoids (Fig. 1g). The absolute contents of geraniol, muurolene, TMTT, citronellal, and citronellol were comparatively high, with VOCs in leaves showing higher concentrations than those in shoots and roots (Fig. 1h).

The TPSs function as key metabolic regulators in the biosynthesis of diverse plant terpenoids [4]. To investigate TPSs responsible for volatile monoterpenes in the *Cymbopogon* genome, we examined reported genes participating in the citronelloid biosynthesis pathways of other species [5] (Table S3). A total of 82 and 86 *TPSs* were expressed in different tissues of *C. winterianus* and *C. distans*, respectively (Table S4). Phylogenetic tree analysis of *CwTPS* genes in *C. winterianus* and *C. distans* belongs to the TPS-a, TPS-b and TPS-g, and TPS-e/f subfamilies, respectively (Figs 1i and S3). Additionally, the enzymatic modifications of geraniol across various plant species have been characterized. The cloning of dehydrogenases responsible for the geraniol–citral (a mixture of geranial and neral) conversion have been reported in alcohol dehydrogenase (ADH) [1]. Citral is capable of being further reduced to citronellal by reductases, which have been identified as geranial reductase, 12-oxophytodienoate reductase (OPR), and citronellal reductase (CAR) [5]. In this study, such citronelloid-biogenesis-related genes were identified and analyzed in *C. winterianus* and *C. distans* (Figs S3–S7; Table S4). Understanding the relationship between chemical diversity and genetic information is essential for elucidating biosynthetic pathways. Subsequently, we carried out an association analysis between the metabolome and transcriptome of *C. winterianus* to identify candidate genes participating in the citronelloid biosynthesis pathway. We identified 19 differentially expressed genes (DEGs) that showed strong positive correlation with citronelloid content, respectively (*R* > 0.99), including seven *CwTPS*, five *CwADH*, two *CwOPR,* and five *CwCAR* genes were identified (Table S5). The expression heatmap demonstrated that these genes were expressed at higher level in *C. winterianus* leaves compared to *C. distans* leaves among the same clades (Figs S3–S7). The results of quantitative reverse transcription PCR (RT-qPCR) were further confirmed and identified that these genes showed higher expression in leaves than that in shoots and roots of *C. winterianus* (Fig. S8). In conclusion, we characterized the candidate genes involved in citronelloid biosynthesis in *C. winterianus*.

Lastly, we conducted single-cell RNA sequencing of nuclei from *C. winterianus* leaves using two biological replicates, generating 135 Gb data. We integrated the datasets using UMAP, resulting in 12 transcriptionally distinct cell clusters (Fig. 1j; Tables S6 and S7). The marker genes from *Arabidopsis*, maize, tomato, and rice were used to determine the cellular identities of *C. winterianus* clusters via orthologous genes (Table S8). To detect DEGs among distinct cell clusters will contribute to explaining and understanding specialized metabolism in *C. winterianus* leaves. Nineteen candidate genes were identified above through integrated transcriptome and metabolome data sets. Notably, four candidate genes showed no expression in any single cells, highlighting the importance of conducting transcriptomic analysis at both bulk tissue and single-cell levels. It suggested that rare or low-abundance transcripts in scRNA-seq may be missed due to technical limitations in sequencing depth as well as differences in gene alignment and quantification methods. Our scRNA-seq data indicated distinct expression patterns of genes for citronelloid biosynthesis (Fig. 1k; Table S9), with most candidate *CwTPS* genes highly expressed in mesophyll cells (cluster 2), suggesting that mesophyll cells might be a crucial cell type for citronelloid biosynthesis. Furthermore, dehydrogenase and reductase enzymes (ADH, OPR, and CAR) showed distinct expression patterns among cell types, potentially playing an important role in ensuring citronelloid diversity in *C. winterianus*. These results imply that expression of genes related to citronelloid biosynthesis occurs in distinct cell types, potentially contributing to pathway compartmentation. To elucidate the molecular mechanisms underlying the contribution of candidate genes to citronelloid biosynthesis, *in vitro* enzyme activity assays are warranted. First, we will overexpress the candidate genes in the heterologous expression system. Subsequently, absolute quantification methods such as isotope-labeled standards will be performed to characterize the functions of candidate genes involved in citronelloid biosynthesis.

In summary, this study unveils the first high-quality genome assemblies in *Cymbopogon*. The research identifies candidate genes involved in citronelloid biosynthesis, drawing from genomic, transcriptomic, and metabolic resources. Our integrative analysis of transcriptomics and metabolomics data from *C. winterianus* led to the identification of candidate homolog genes (*CwTPS*, *CwADH,* and *CwCAR*) potentially involved in citronelloid biosynthesis. Additionally, single-cell RNA sequencing revealed distinct cellular locations of these candidate genes. This study not only contributes to understanding evolution of citronelloid biosynthetic pathways within Poaceae family, but also provides abundant cellular and genetic resources for further molecular biology research on *C. winterianus* and other plant species.

## Supplementary Material

Web_Material_uhaf287

## Data Availability

All high-throughput sequencing datasets have been deposited in the Genome Sequence Archive at the National Genomics Data Center, China National Center for Bioinformation (BioProject ID: PRJCA038074). All supplementary metabolomic and transcriptomic data is available in Figshare (https://doi.org/10.6084/m9.figshare.29432150).
