# Supplementary material for: Haplotype-resolved genome of a citronella provides insights into the evolution of citronelloid biogenesis pathway
Source: Hortic Res. 2025 Oct 16;13(2):uhaf287. doi: 10.1093/hr/uhaf287 (PMC12893864; doi:10.1093/hr/uhaf287)
Supplement: Web_Material_uhaf287 [file web_material_uhaf287.zip › Figures Supplementary 2.docx]

**Supporting information**

**Article:** Haplotype resolved genome of a citronella provides insights into the evolution of citronelloid biogenesis pathway

Hai He^1,2^, Zemian Lin^1,2^, Zanchen Zhou^1^, Pinhao Chen^1^, Lifeng Xia^1^, Hao Li^1^, Yu Zhang^1,3,*^

^1^School of Agriculture and Biotechnology, Sun Yat-Sen University, Shenzhen, China

^2^These authors contributed equally

^3^ Lead contact

^*^Corresponding author:

zhangy2526@mail.sysu.edu.cn, ORCID: 0000-0001-6547-6243

**Contents**:

Figures. S1-S8


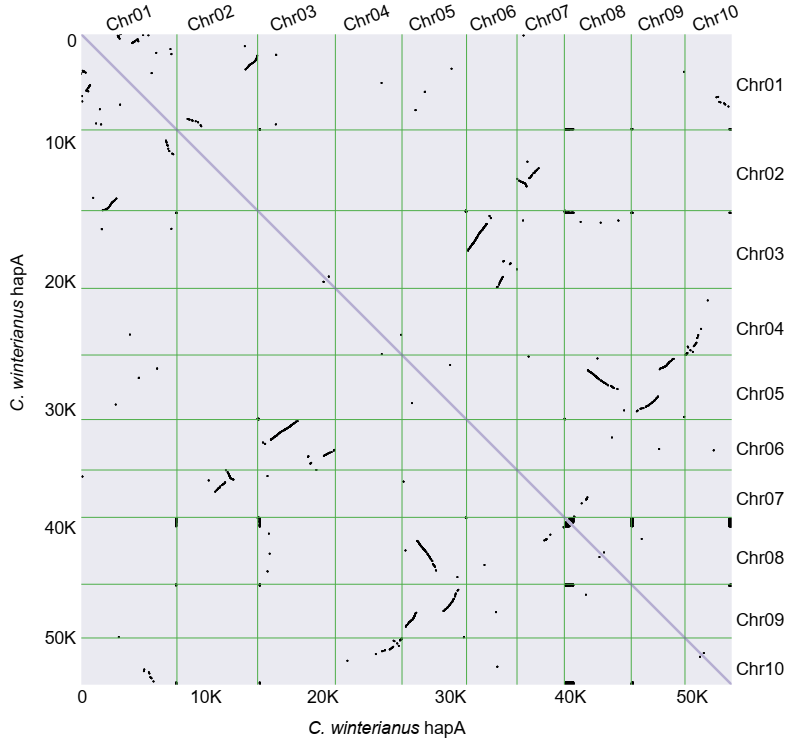


**Figure S1.** **Intra-genomic comparison in the haplotype A subgenome of C. winterianus.** Both X-axis and Y-axis represent 3,907 genes in the chromosomal order, and the best matching gene pairs are plotted.


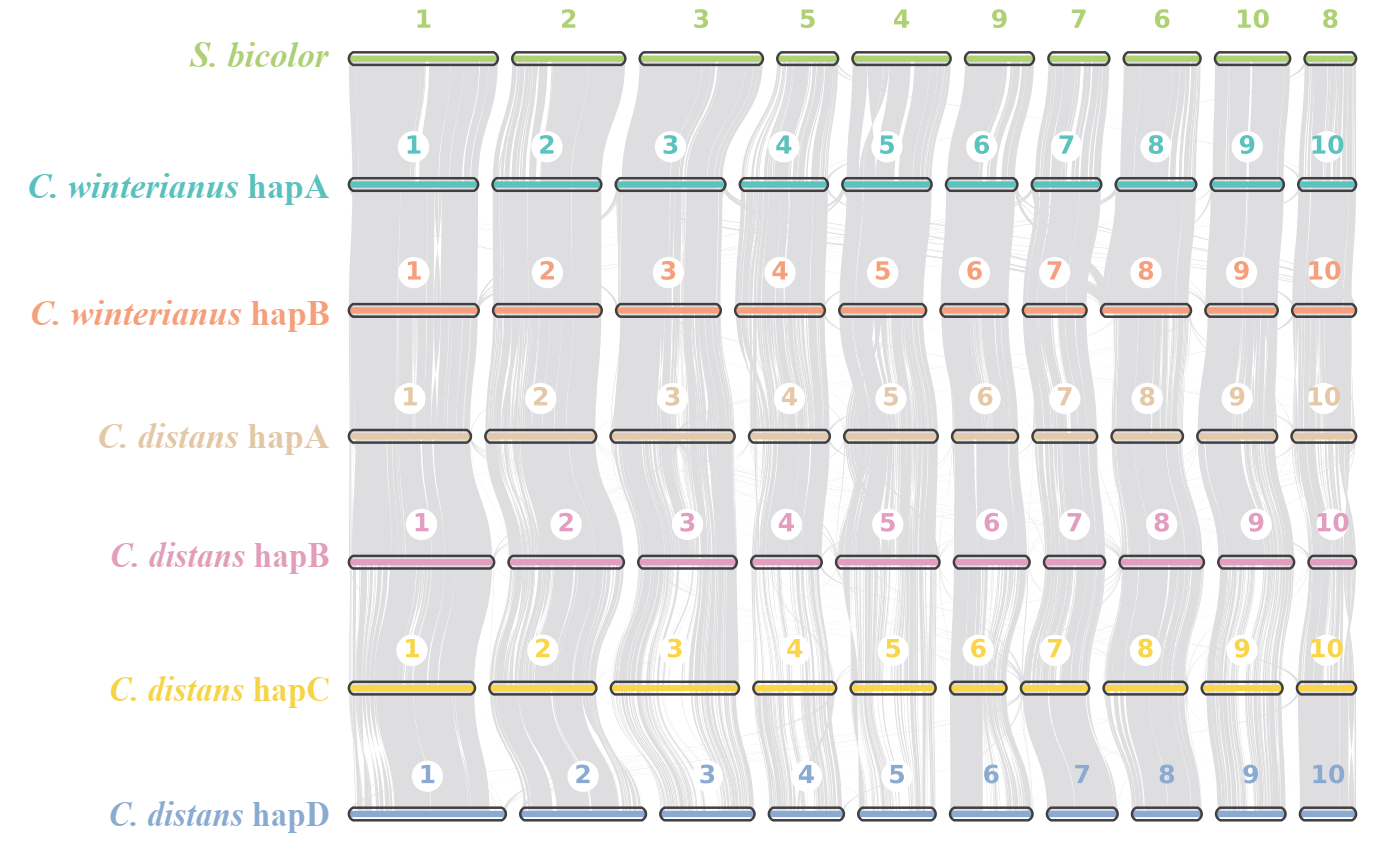


**Figure S2. Collinearity analysis among the genome of *S. bicolor*, two subgenomes of *C. winterianus*, and the four** **subgenomes of *C. distans*.**


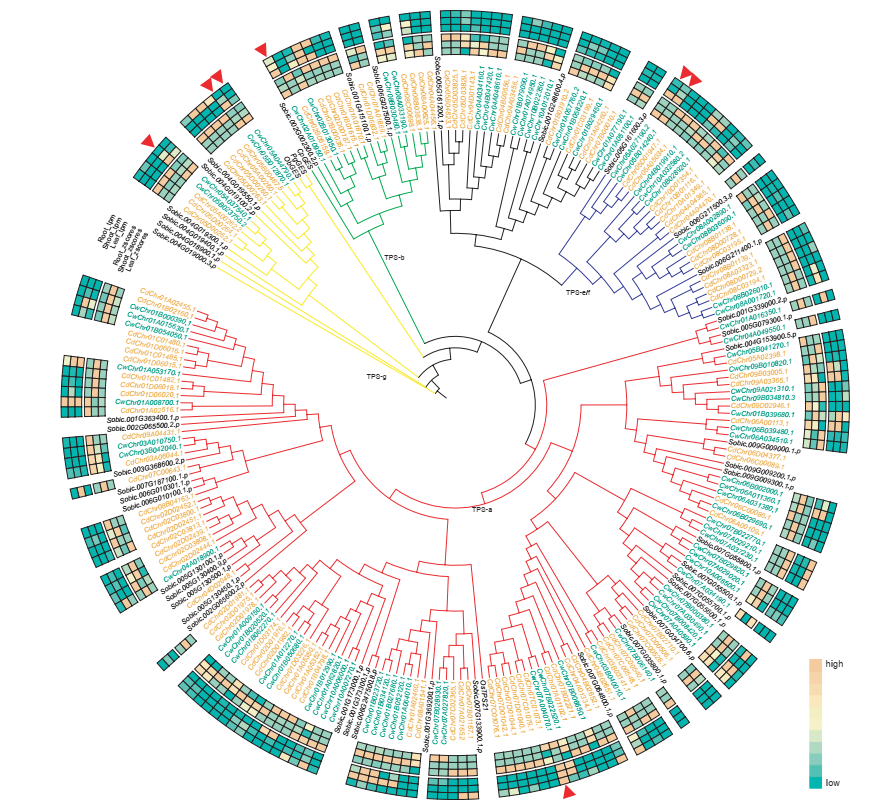


**Figure S3. Phylogenetic trees of the TPSs involved in the terpenoids biosynthetic pathway.** The branches colored in red, green, yellow, and blue represent members of TPSa, TPSb, TPSg and TPSe/f, respectively. The black branches denote the other TPS families. The circle display gene expression (TPM on the outside, z-score on the inside) in leaves, shoots, and roots of *C. winterianus* and *C. distans*. Red triangles indicate candidate genes.


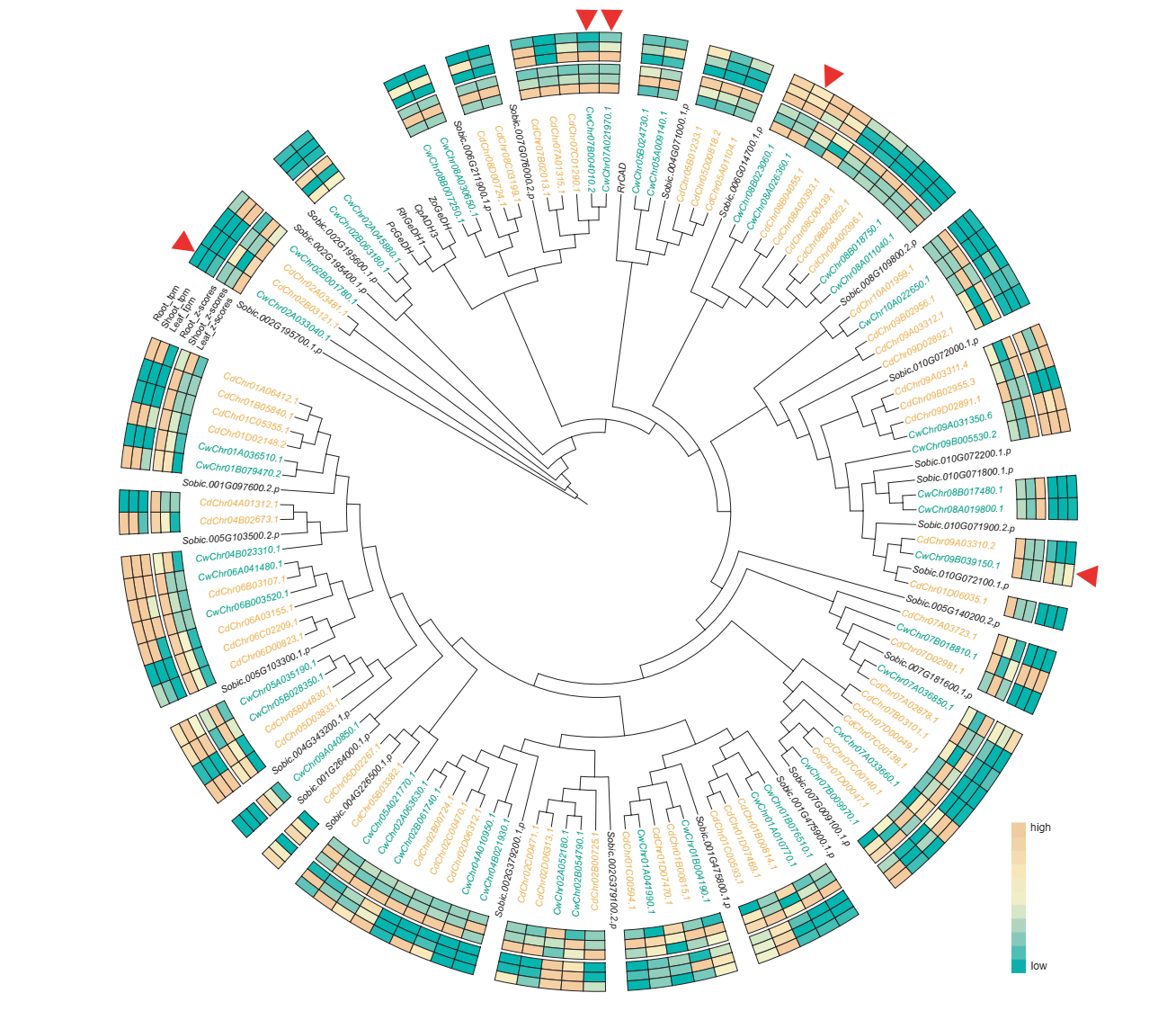


**Figure S4. Phylogenetic trees of the ADH/GeDH/CAD involved in the terpenoids biosynthetic pathway.** The circle display gene expression (TPM on the outside, z-score on the inside) in leaves, shoots, and roots of *C. winterianus* and *C. distans*. Red triangles indicate candidate genes.


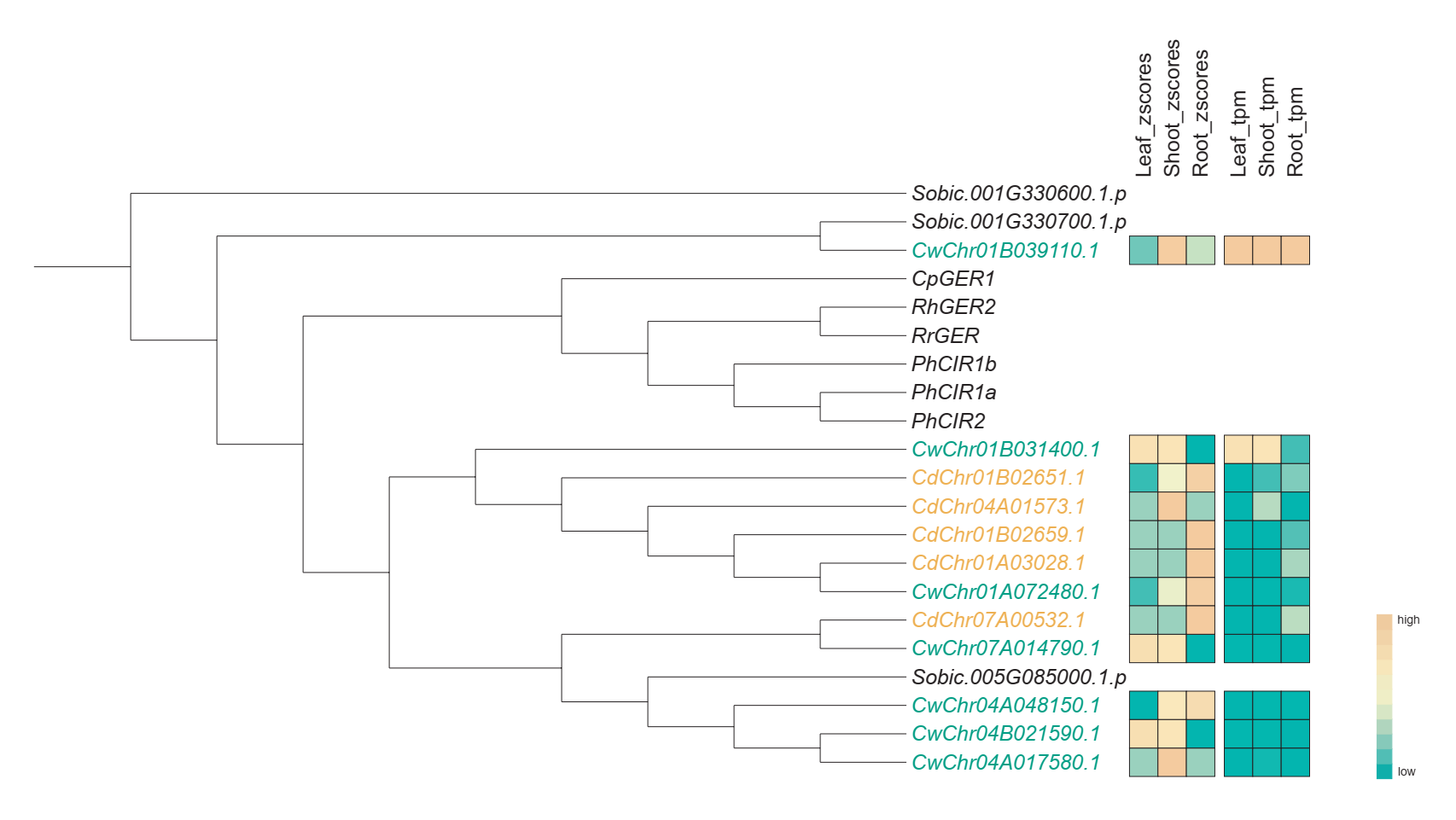


**Figure S5. Phylogenetic trees of the GER/CIR involved in the terpenoids biosynthetic pathway.** The right heatmap display gene expression (TPM on the right, z-score on the left) in leaves, shoots, and roots of *C. winterianus* and *C. distans*.


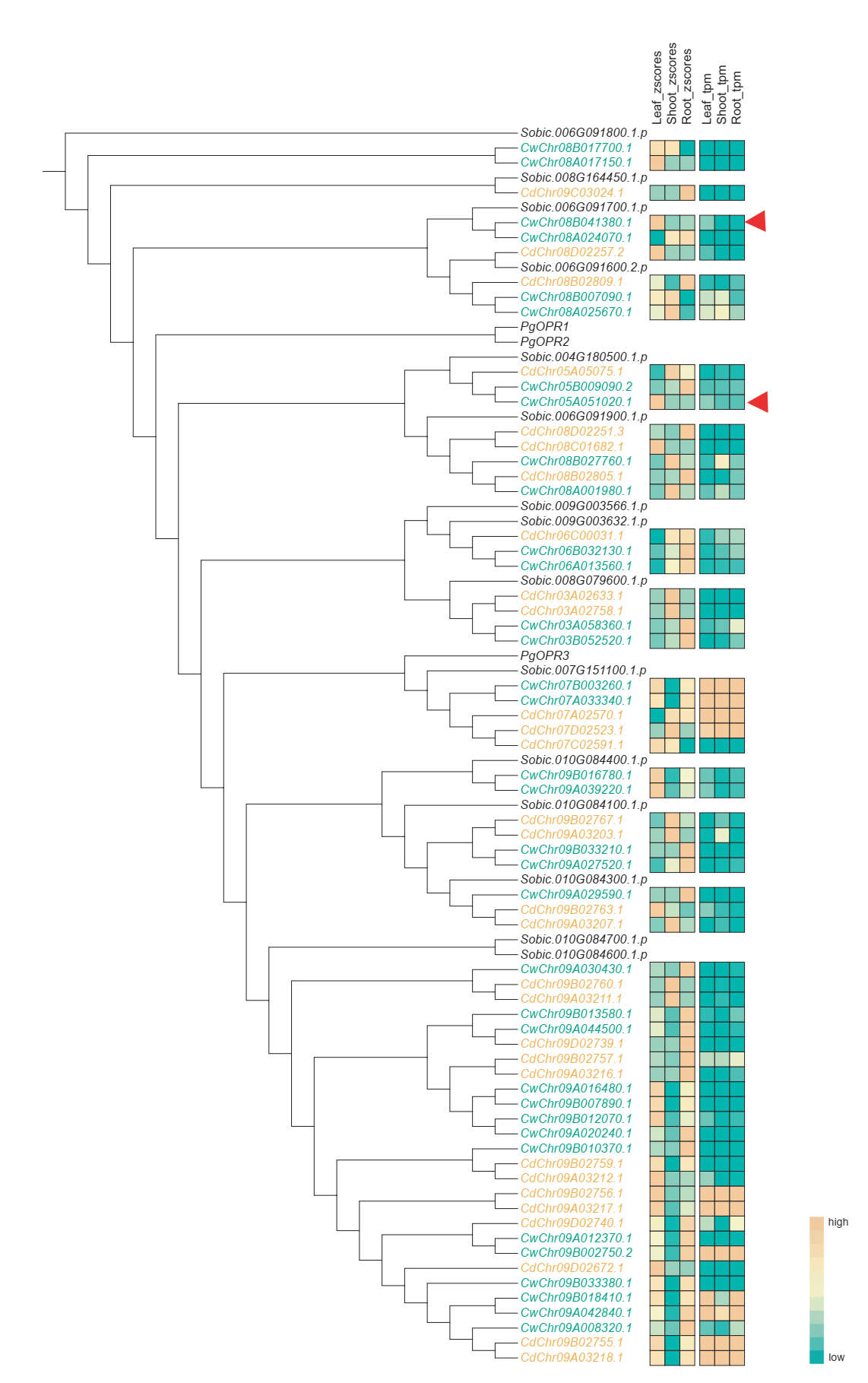


**Figure S6. Phylogenetic trees of the OPR involved in the terpenoids biosynthetic pathway.** The right heatmap display gene expression (TPM on the right, z-score on the left) in leaves, shoots, and roots of *C. winterianus* and *C. distans*. Red triangles indicate candidate genes.


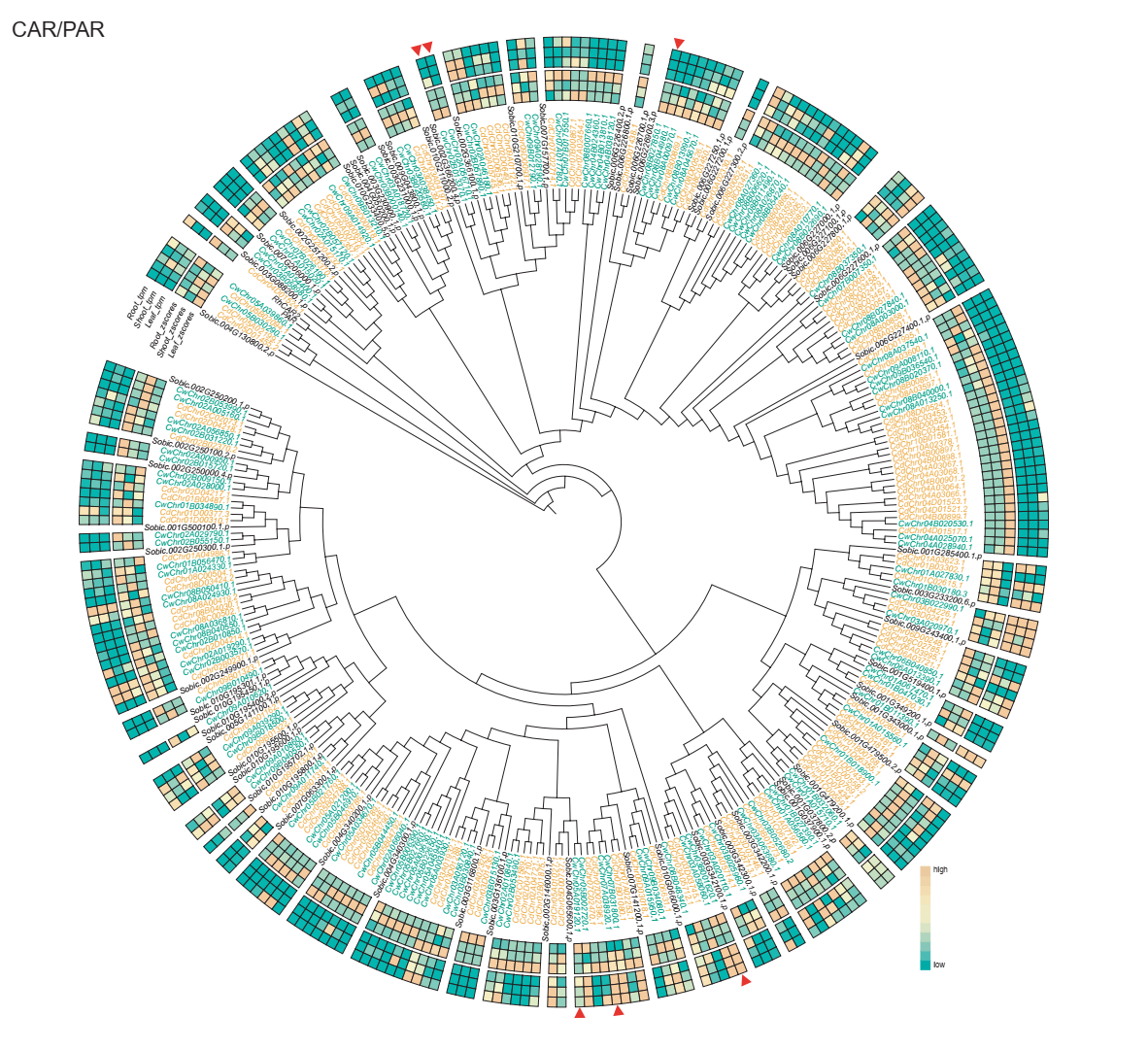


**Figure S7. Phylogenetic trees of the PAR/CAR involved in the terpenoids biosynthetic pathway.** The circle display gene expression (TPM on the outside, z-score on the inside) in leaves, shoots, and roots of *C. winterianus* and *C. distans*. Red triangles indicate candidate genes.


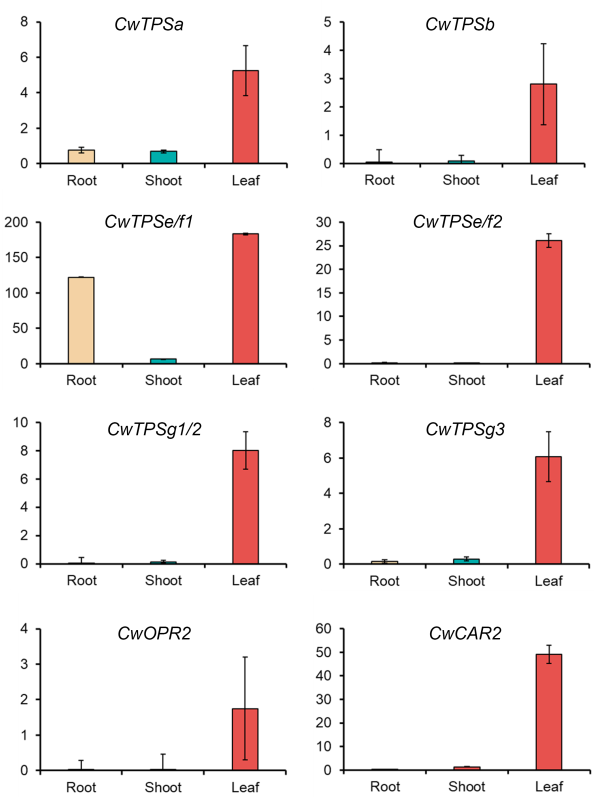


**Figure S8. Relative expression levels of candidate genes in root, shoot and leaves of *C. winterianus* by RT-qPCR.** *Actin* was used as internal reference genes. Error bars represent standard error of the mean.
