# Supplementary material for: Haplotype-resolved genome of a citronella provides insights into the evolution of citronelloid biogenesis pathway
Source: Hortic Res. 2025 Oct 16;13(2):uhaf287. doi: 10.1093/hr/uhaf287 (PMC12893864; doi:10.1093/hr/uhaf287)
Supplement: Web_Material_uhaf287 [file web_material_uhaf287.zip › Materials and methods 2.docx]

**Materials and methods**

**Plant materials and growth conditions**

The *C. winterinus* and *C. distans* were cultivated at 25°C in a greenhouse at Sun Yat-sen University' School of Agriculture and Biotechnology in Guangdong, China. The photoperiod consists of 12 hours light and 12 hours dark. Samples were collected at the stage of fully developed vegetative stage, when plants were fully tillering of shoots. The research materials were separated into three parts: leaves, shoots, and roots (Fig. 1a and 1b). The samples were promptly immersed in liquid nitrogen for freezing and kept at -80°C until for analysis.

**Analysis of volatile compounds**

The volatile compounds were gather through HS-SPME and examined by GC–MS^1^. An Agilent Model 8890 GC along with a 7000D mass spectrometer from Agilent, which was fitted with a 30 m × 0.25 mm × 0.25 μm DB-5MS capillary column, was employed for analysis. Differential metabolites among tissues were identified based on the following criteria: variable importance in the project being ≥ 1, the absolute value of the |log2fold change| being ≥ 2, and the P-value being < 0.05. For transcriptome and metabolome data analysis, Pearson's correlation coefficients (PCC) among the DEGs and differential metabolites were analyzed, with strong correlation conditions defined as |PCC| ≥ 0.8 and *P*-value < 0.05. Three types of *C. winterinus* were collected for metabolomics determination.

**Bulk RNA-seq sequencing and data analysis**

Total RNA was obtained from diverse tissue of *C. winterinus* and *C. distans* with TRIzol, following the manufacturer’s instructions for three biological replicates. The qualified libraries of *C. winterinus* were combined and sequenced using an Illumina X Plus, while *C. distans* was sequenced on an Illumina NovaSeq 6000 platform, with each sample generating 6 GB of raw data. The clean reads were mapped using HISAT2 (v2.2.1) with default parameters. DEGs were identified using DEGseq2 (v1.40.2) between different tissues with |log2FC| ≥1 and padj ≤ 0.05. Functional annotations of DEGs were performed using the KEGG databases. Leaf, shoot, and root tissues from *winterinus* and *C. distans* were gathered for transcriptome sequencing.

**Genome assembly and assessment**

The fresh leaves of *C. winterinus* and *C. distans* plants were employed for the isolation of genomic DNA through the CTAB method. HiFi sequencing was carried out by using the Sequel Ⅱ system (PacBio). Hi-C sequencing libraries were sequenced on the Illumina HiSeq-2500 platform. GenomeScope2^2^ was utilized to assess the genome size of *C. winterinus*. The heterozygosity and repeat rate of the *C. winterinus* genomes were calculated via the *K*-mer method with Jellyfish (v2.2.10)^3^. HiFiasm (v0.16.1)^4^ was employed to obtain draft genomes using the HiFi sequencing reads and Hi-C sequencing data of *C. winterinus* and *C. distans*. Subsequently, the contiguous assembly of *C. winterinus* and *C. distans* genomes was performed using the 3D-DNA (v2.2.10)^5^. The Hi-C sequencing reads were initially aligned to the draft genome assembly using Jucier (v1.6), and manual review of the candidate assemblies were conducted using Juicebox (v2.17.00)^6^. Finally, the chromosome-scale assemblies were further integrated by 3D-DNA (v201013)^7^ with default parameters and evaluated using BUSCO (v5.4.5)^8^.

**Repeat annotation genome annotation**

The extensive *de novo* TE annotator (EDTA, v2.1.3) was utilized to generate a TE library for the *de novo* identification. After these TE sequences were masked, Evidence Modeler (v1.1.1) was employed to predict high-quality protein-coding genes using three approaches, ab initio gene predictions, transcription evidence, and predictions based on homology. Augustus (v3.5.0)^9^ was used for ab initio gene prediction Regarding the transcript-based predictions, RNA-seq data sourced from various tissues (leaf, shoot, root) were assembled using HISAT2 (v2.2.1)^10^, stringtie2 (v2.2.1), and all the assembled transcript ORFs were predicted by means of Trans-Decoder (v5.7.0). Homology-based annotation was conducted using proteins from five species: *Oryza sativa*, *Arabidopsis thaliana*, *Zea mays*, *Brachypodium distachyon*, and *Setaria viridis* with the GeMoMa (v1.9). To enhance the accuracy of the annotation, we integrated and updated the gene prediction results using Evidence Modeler (v1.1.1) and PASA (v2.5.3). BUSCO (v5.4.5) was employed to assess quality of the prediction.

**Functional Annotation**

After genome structure annotation was finished, three strategies were employed to predict the gene function. The eggnog-mapper (v2.1.12) was utilized to look for homologous genes in the eggNOG database, which contains Gene Ontology (GO) and Kyoto Encyclopedia of Genes and Genomes (KEGG). Subsequently, four protein databases: Swiss-Prot protein knowledgebase (Swiss-prot), translated EMBL nucleotide sequence database (TrEMBL), non-redundant protein sequence database (NR) and TAIR10 protein database were applied to conduct the alignment of protein-coding genes using DIAMOND (v2.1.8). Lastly, InterProScan (v5.61-93.0) was employed to annotate the gene motifs and domains.

**Analysis of terpenoid biosynthesis genes**

Genes participating in terpenoid biosynthesis via the MVA and MEP pathways were identified in *C. winterinus*, *C. distans*, and other species based on the genome sequence similarity and annotation with *Arabidopsis* or rose proteins^1,11^. TPS members of *C. winterinus* and *C. distans* were classified according to their the phylogenetic relationships with previously established classifications in *Arabidopsis* and rice^12,13^. OrthoFinder (v2.5.5) was used to identify Orthologous groups. The phylogenetic tree was built with iqtree (v2.3.4), and visualized using iTOL.

**Genome evolution analysis**

To investigate the evolution of the *Cymbopogon* genomes, we conducted a comparative genomic analysis of *C. winterinus*, *C. distans*, *C. reinhardtii*, *P. patens*, *S. moellendorffii*, *C. richardii*, *T. plicata*, *A. trichopoda*, *R. rugosa*, *A. thaliana*, *O. thomaeum*, *C. sinensis*, *C. clementina*, *A. officinalis*, *O. sativa*, *Z. officinale*, *P. latifolius*, *B. distachyon*, *Z. mays*, *S. bicolor*, and *C. citriodora* using OrthoFinder (v2.5.5). Single-copy gene families underwent multiple sequence alignments using MAFFT (v7.520) with the "-auto" parameter. The alignments were then trimmed by Gblocks (v0.91b), and a phylogenetic tree was constructed using iqtree (v2.3.4). The divergence time among 21 plants was analyzed using the MCMCTree program of the PAML package based on the estimated divergence times of *C. clementina*-*C. sinensis* (1.5-5.7 Mya) from the TimeTree database. For WGD analysis, the syntenic regions between and within *C. winterinus*, *C. distans*, *A. thaliana*, and *S. bicolor* were identified using MCscanX (v1.0.0)^14^ based on all-to-all BLASTP results. *Ks* was computed by mean of KaKs Calculator with the YN method^15^. The R package was used to visualize the *Ks* distribution map. Phylogenetic analysis was performed based on highly conserved nuclear low-copy genes^16^. The divergence time between *Amborella trichopoda* and *Thuja plicata* was used for calibration. The tree was reconstructed using BEAST X (v.10.5.0), and convergence was confirmed with Tracer (v1.7.2, all effective sample sizes > 200). The resulting chronogram, visualized using FigTree. A total of 3,907 gene sequences from the haplotype A genome of *C. winterianus*, ordered by their chromosomal number, were subjected to self-comparison using BLAST (v.2.12.0). Intra-genomic comparisons and chromosome scale dot-plot were constructed using the Python version of MCscanX. Genome collinearity analysis and their visualizations were also performed with this tool.

**scRNA-seq library construction and sequencing**

The nuclei of *C. winterinus* leaves were isolated, and libraries were constructed with the Chromium Next Single Cell 3′ GEM Library and Gel Bead Kit v.3 according to the manufacturer's instructions. Subsequently, sequencing was performed on an Illumina NovaSeq X plus. For the Cell Ranger pipeline, Cell Ranger (v8.0.1) was employed to map reads to the reference *C. winterinus* genome using default parameters. We initially merged two biological replicates and performed standard preprocessing steps to remove low-quality cells using the R package Seurat (v.5.0.3). Specifically, cells expressing fewer than 250 genes and less than 500 UMIs were excluded from the analysis. Cells with mitochondrial and chloroplast expression below 5% were retained. The gene expression values were calculated by carrying out data normalization with the NormalizeData function in Seurat. The RunHarmony (v1.2.3) function was utilized to reduce the batch effect.

**Cell clustering and annotation**

Utilizing the integrated data assay, principal-component analysis (PCA) was conducted to reduce the dimensionality using the default function in Seurat (v.5.0.3). The RunUMAP function was used to visualize cell clusters (reduction = pca, dims = 1:30). Cell clusters were recognized by applying the FindNeighbors function (with reduction = pca, dims = 1:30), and FindClusters function at a resolution of 0.2. To assign putative cell types to each cluster, FindAllMarkers was used to identify positive markers by comparing each cluster with all other clusters. A logfc.threshold of 0.25 was set as the threshold for significantly different expression.

**Differential gene expression and marker identification**

To identify different cell types, we utilized established marker genes from rice and *Arabidopsis* to annotate *C. winterinus* cell types. The list of marker gene was obtained from Plant Cell Marker Database^17^, and additional *Arabidopsis* marker genes were compiled from published scources^18,19^. The rice, *Arabidopsis*, tomato, and maize marker genes were used as the query sequences to identify the homologous genes in *C. winterinus* from protein clusters generated by BLAST (v.2.12.0)^20,21^. The hits with the highest scores were selected and labeled as corresponding *C. winterinus* cell types. To define DEGs in various cell types, the FindMarkers function was used, setting the parameters logfc.threshold to 0.1 and min.pct to 0.1. The intersections of DEGs among samples were visualized by using the ggplot2 (v3.5.0).

**RT-qPCR assays**

RT-qPCR assays were performed in a 10 μL reaction mixture containing 5 μL of one step SYBR Green Mix (Vazyme, China) with three technical and three biological replicates. Primers were listed in Table S10. Relative gene expressions were determined using the 2^-∆CT^ formula.

**References**

1. Li H, Li Y, Yan H, Bao T, Shan X, Caissard JC, Zhang L, Fang H, Bai X, Zhang J, Wang Z, Wang M, Guan Q, Cai M, Ning G, Jia X, Boachon B, Baudino S, Gao X. (2024). The complexity of volatile terpene biosynthesis in roses: Particular insights into β-citronellol production. Plant Physiol. *196*, 1908–1922.
2. Vurture, G.W., Sedlazeck, F.J., Nattestad, M., Underwood, C.J., Fang, H., Gurtowski, J., and Schatz, M.C. (2017). GenomeScope: fast reference-free genome profiling from short reads. Bioinformatics *33*, 2202–2204.
3. Marçais, G., and Kingsford, C. (2011). A fast, lock-free approach for efficient parallel counting of occurrences of *k* -mers. Bioinformatics *27*, 764–770.
4. Cheng, H., Concepcion, G.T., Feng, X., Zhang, H., and Li, H. (2021). Haplotype-resolved *de novo* assembly using phased assembly graphs with hifiasm. Nat Methods *18*, 170–175.
5. Van Berkum, N.L., Lieberman-Aiden, E., Williams, L., Imakaev, M., Gnirke, A., Mirny, L.A., Dekker, J., and Lander, E.S. (2010). Hi-C: A method to study the three-dimensional architecture of genomes. J. Vis. Exp. *39*, e1869.
6. Robinson, J.T., Turner, D., Durand, N.C., Thorvaldsdóttir, H., Mesirov, J.P., and Aiden, E.L. (2018). Juicebox.js provides a cloud-based visualization system for Hi-C data. Cell Syst. *6*, 256-258.
7. Dudchenko, O., Batra, S.S., Omer, A.D., Nyquist, S.K., Hoeger, M., Durand, N.C., Shamim, M.S., Machol, I., Lander, E.S., Aiden, A.P., et al. (2017). *De novo* assembly of the *Aedes aegypti* genome using Hi-C yields chromosome-length scaffolds. Science. *356*, 92–95.
8. Simão, F.A., Waterhouse, R.M., Ioannidis, P., Kriventseva, E.V., and Zdobnov, E.M. (2015). BUSCO: assessing genome assembly and annotation completeness with single-copy orthologs. Bioinformatics. *31*, 3210–3212.
9. Keller, O., Kollmar, M., Stanke, M., and Waack, S. (2011). A novel hybrid gene prediction method employing protein multiple sequence alignments. Bioinformatics. *27*, 757–763.
10. Pertea, M., Kim, D., Pertea, G.M., Leek, J.T., and Salzberg, S.L. (2016). Transcript-level expression analysis of RNA-seq experiments with HISAT, StringTie and Ballgown. Nat Protoc. *11*, 1650–1667.
11. Vranová, E., Coman, D., and Gruissem, W. (2013). Network analysis of the MVA and MEP pathways for isoprenoid synthesis. Annu. Rev. Plant Biol. *64*, 665–700.
12. Aubourg, S., Lecharny, A., and Bohlmann, J. (2002). Genomic analysis of the terpenoid synthase (*AtTPS*) gene family of *Arabidopsis thaliana*. Mol Gen Genomics. *267*, 730–745.
13. Sun, Y., Zhang, P., Kou, D., Han, Y., Fang, J., Ni, J., Jiang, B., Wang, X., Zhang, Y., Wang, W., et al. (2022). Terpene synthases in rice pan-genome and their responses to *Chilo suppressalis* larvae infesting. Front. Plant Sci. *13*, 905982.
14. Wang, Y., Tang, H., DeBarry, J.D., Tan, X., Li, J., Wang, X., Lee, T. -h., Jin, H., Marler, B., Guo, H., et al. (2012). MCScanX: a toolkit for detection and evolutionary analysis of gene synteny and collinearity. Nucleic Acids Res. *40*, e49.
15. Wang, D., Zhang, Y., Zhang, Z., Zhu, J., and Yu, J. (2010). KaKs_Calculator 2.0: A toolkit incorporating gamma-series methods and sliding window strategies. Genom Proteom Bioinf. *8*, 77–80.
16. Zhang, N., Zeng, L., Shan, H., and Ma, H. (2012). Highly conserved low-copy nuclear genes as effective markers for phylogenetic analyses in angiosperms. New Phytol. *195*, 923–937.
17. Jin, J., Lu, P., Xu, Y., Tao, J., Li, Z., Wang, S., Yu, S., Wang, C., Xie, X., Gao, J., et al. (2022). PCMDB: a curated and comprehensive resource of plant cell markers. Nucleic Acids Res. *50*, D1448–D1455
18. Sun, X., Feng, D., Liu, M., Qin, R., Li, Y., Lu, Y., Zhang, X., Wang, Y., Shen, S., Ma, W., et al. (2022). Single-cell transcriptome reveals dominant subgenome expression and transcriptional response to heat stress in Chinese cabbage. Genome Biol *23*, 262.
19. Bai, Y., Liu, H., Lyu, H., Su, L., Xiong, J., and Cheng, Z.-M. (2022). Development of a single-cell atlas for woodland strawberry (*Fragaria vesca*) leaves during early *Botrytis cinerea* infection using single-cell RNA-seq. Horticulture Research *9*, uhab055.
20. Yue, H., Chen, G., Zhang, Z., Guo, Z., Zhang, Z., Zhang, S., Turlings, T.C.J., Zhou, X., Peng, J., Gao, Y., Zhang, D., Shi, X., Liu, Y.. (2024) Single-cell transcriptome landscape elucidates the cellular and developmental responses to tomato chlorosis virus infection in tomato leaf. Plant Cell Environ. *47*:2660–2674.
21. Bezrutczyk, M., Zöllner, N.R., Kruse, C.P.S., Hartwig, T., Lautwein, T., Köhrer, K., Frommer, W.B., Kim, J.Y. (2021) Evidence for phloem loading via the abaxial bundle sheath cells in maize leaves. Plant Cell. *33*:531–547.
